# Mechanical and Electromagnetic Wave Absorption Performance of Carbonyl Iron Powder-Modified Nonwoven Materials

**DOI:** 10.3390/ma16237403

**Published:** 2023-11-28

**Authors:** Wenyan Gu, Jiang Shi, Tianwen Pang, Qilong Sun, Qi Jia, Jiajia Hu, Jiaqiao Zhang

**Affiliations:** 1 School of Textile and Clothing, Nantong University, Nantong 226019, China; gu.wy@ntu.edu.cn (W.G.); 2215320009@stmail.ntu.edu.cn (J.S.); 2315310019@stmail.ntu.edu.cn (T.P.); sunqilong001@ntu.edu.cn (Q.S.); 1915110134@stmail.ntu.edu.cn (Q.J.); 1915110148@stmail.ntu.edu.cn (J.H.); 2 School of Mechanical Engineering, Southeast University, Nanjing 211189, China

**Keywords:** carbonyl iron, nonwoven fabric, electromagnetic wave absorption, composites

## Abstract

In order to develop carbonyl iron-enhanced electromagnetic wave-absorbing composites, this paper utilizes two different morphologies of carbonyl iron powder (CIP), spherical and flake-like, which are blended with aqueous polyurethane (PU) in three different ratios to prepare impregnating solutions. Polyester (PET) needle-punched nonwoven materials are impregnated with these solutions to produce electromagnetic wave-absorbing composites. First, electromagnetic parameters of the two CIP particle types, spherical carbonyl iron (SCIP) and flake-like carbonyl iron (FCIP), are tested with the coaxial method, followed by calculation of the results of their electromagnetic wave absorption performance. Next, the composites are subjected to microscopic morphology observation, tensile testing, and arched frame method electromagnetic wave absorption performance testing. The results indicate that the microwave absorption performance of FCIP is significantly better than that of SCIP. The minimum reflection loss value for F3, a kind of FCIP-modified nonwoven fabric, at the thickness of 1 mm, at 18 GHz is −17 dB. This value is even better than the calculated RL value of CIP at the thickness of 1 mm. The anisotropic shape of flake-like magnetic materials is further strengthened when adhering to the surface of PET fiber material. Additionally, the modified composites with carbonyl iron exhibit higher tensile strength compared with pure PET. The addition of fibrous skeletal materials is expected to enhance the impedance matching of flake-like magnetic particles, forming a wearable and microwave-absorbing composite.

## 1. Introduction

With the rapid development of wireless communication technology, electromagnetic wave applications are increasingly integrated into human production and daily life. They play a significant role in information transmission and productivity improvement while also raising concerns about adverse effects on human health, such as thermal effects [1] and electromagnetic radiation. Electromagnetic wave-absorbing materials, through the loss of specific frequency electromagnetic waves [2], offer a solution to mitigate the negative impacts of electromagnetic wave applications [3].

Carbonyl iron powder (CIP) is a typical magnetic loss electromagnetic wave absorbing filler known for its advantages including good thermal stability, high magnetic permeability, high saturation magnetization, and low cost [4]. However, CIP tends to agglomerate, making it challenging to disperse uniformly. It also has poor dielectric properties and is susceptible to corrosion and oxidation, which can affect its effectiveness in microwave absorption. To overcome these drawbacks, researchers have modified the surface of CIP particles to create core–shell structured composites. For example, Guo [5] prepared core–shell structured CIP@30% epoxy resin (EP) using in situ polymerization, achieving a minimum reflection loss (RL_min_) of −5.4 dB at a thickness of 16.1 mm and a broad effective absorption bandwidth (EAB, RL < −10 dB) of 2.2 GHz. Wang et al. [6] used the sol–gel method to prepare core–shell CIP@SiO_2_, improving the impedance matching of CIP, and achieved an EAB of up to 7.12 GHz (10.88~18.00 GHz) at a thickness of 1.5 mm. Zu et al. [7] employed a one-step polymerization method to produce FCIP@EP with a higher magnetic threshold, resulting in a 7.63 GHz EAB for a 1.5 mm thick composite at 23 vol%. Additionally, researchers have attempted to incorporate CIP into organic matrices to maximize performance. For instance, He et al. [8] used a rotating magnetic field and various cutting methods to create flake-like CIP@SiO_2_ particle/polyurethane (PU), finding significantly improved microwave absorption properties compared with the original CIP. Semenenko et al. [9] uniformly dispersed CIP in an organosilicon polymer solution, forming a high-viscosity suspension, and used air spray painting techniques to produce uniformly thick planar materials. Using the free space method in the 3~39 GHz range to measure the normal incidence reflectivity of metal-backed composites, they found that it is possible to produce single-layer radar absorbing materials with thicknesses less than 1.5 mm, with deep and wide minimum values of normal incidence reflectivity (less than −20 dB) in the 10 to 30 GHz range. Jang et al. [10] dispersed different weight ratios of CIP magnetic fillers in a polydimethylsiloxane (PDMS) matrix and observed that RL_min_ increased as CIP filler weight increased from 46% to 72%. With a thickness of 1.5 mm, they achieved a high EAB of 6.8 GHz, with RL_min_ reaching −27.5 dB at 14.6 GHz. Zhou et al. [11] hybridized carbonyl iron and Ti_3_SiC_2_ powders with epoxy resin and polyimide resin to create microwave-absorbing composites. They found that increasing Ti_3_SiC_2_ content or reducing Ti_3_SiC_2_ particle size significantly improved the real and imaginary parts of the composite permittivity in the frequency range of 8.2 to 12.4 GHz. When the Ti_3_SiC_2_ content was 7 vol% and the Ti_3_SiC_2_ particle size was <30 μm, the composite achieved an EAB of 3.6 GHz at a thickness of 1.2 mm within the 8.8~12.4 GHz range.

Considering the adverse effects of CIP’s high density on its wave absorption applications and aiming to further enhance impedance matching, researchers have compounded CIP with low-density materials that have good dielectric or magnetic properties [12,13,14]. This is carried out to induce dipole polarization and interface polarization, thereby enhancing the material’s dielectric and magnetic losses and achieving the goals of reducing weight and improving electromagnetic absorption performance. For instance, Jan et al. [15] studied the microwave absorption performance of edge-selectively oxidized graphene (EOG) on carbonyl iron composites (EOG/CIP/PDMS) with different graphene contents. They found that the EOG/CIP/PDMS composite with 5.0 wt.% EOG and 72 wt.% CIP achieved an RL_min_ of −45.37 dB at 10 GHz with an EAB of 4.08 GHz and a thickness of 1.52 mm. Another EOG/CIP composite with 0.5 wt.% EOG and 72 wt.% CIP and a thickness of 1.33 mm reached an RL_min_ of −69.27 dB at 15 GHz with an EAB of 6.47 GHz. Furthermore, CIP and other nanomaterials have been incorporated into special three-dimensional structures, such as gradient structures, honeycomb structures, topological structures, foam structures, multilayer structures, biomimetic structures, and vertically oriented structures, to enhance electromagnetic absorption performance [16,17,18,19,20,21,22,23,24,25,26,27,28]. Honeycomb structure absorbing composites are a significant research focus in this regard. For example, Gong et al. [29] used 3D printing technology to create a flexible honeycomb absorbing structure with carbon fiber/polyamide/carbonyl iron composites. Under high bending angles of up to 150 degrees, the flexible honeycomb achieved an RL_min_ of −47 dB at 16.2 GHz with an EAB of 13.2 GHz, covering the entire C, X, and Ku bands. Lyu [22] used a substrate of carbon black/CIP/basalt fiber/carbon fiber/epoxy resin (CB/CIP/BF/CF/EP) and the vacuum-assisted resin transfer molding (VARTM) process to prepare 3D honeycomb woven electromagnetic absorbing composites. They found that the addition of CB/CIP significantly improved the absorption performance of the composite, and CB/CIP composite absorbers belong to the magnetic loss type. Zhang et al. [19] used basalt and carbon fibers as reinforcements, epoxy resin mixed with carbon black and CIP as the matrix, and the VARTM process to create 3D gradient honeycomb composites. When the thickness was 18.5 mm, the RL_min_ achieved at a 30-degree angle of incidence in the C-band was −47 dB.

In contrast to the aforementioned 3D structures, nonwoven fabric presents a straightforward and cost-effective form of 3D structure, offering advantages such as being lightweight, featuring short manufacturing processes, and exhibiting high design flexibility. Over the past few years, there has been a growing utilization of nonwoven fabrics in the realm of electromagnetic wave-absorbing materials research [30,31,32,33]. Nevertheless, investigations into the application of nonwoven fabrics as structural components in magnetic electromagnetic wave-absorbing composites are limited. This study utilizes aqueous polyurethane as the matrix, employing PET needle-punched nonwoven fabric as a 3D structure to enhance the mechanical strength and flexibility of the composite. Various morphologies of CIP particles serve as functional fillers to regulate electromagnetic parameters and electromagnetic wave absorption performance. Through adjustments in the mass ratio of CIP to PU, the composite’s tensile properties and electromagnetic wave absorption performance are optimized. Ultimately, the composite’s microstructure, tensile strength, and electromagnetic wave absorption performance are characterized.

The main innovation of this paper lies in the quantitative study of the impact of CIP morphology on the electromagnetic wave absorption and mechanical properties of nonwoven fabric. CIP is a kind of traditional magnetic loss type of absorbing material, but its powder state is not conducive to both application and the improvement of absorbing performance. Therefore, it was considered to combine it with PET nonwoven materials with loose fiber aggregation structures, making full use of the rich interfaces of nonwoven materials through fiber interpenetration and entanglement, which interacts with the electromagnetic properties of CIP, to improve the EMW absorption performance of CIP-modified nonwoven materials. Despite SCIP and FCIP sharing the same chemical structure, differences in morphology result in variations in specific surface area, dispersibility, and orientation between the two types of nanoparticles. FCIP typically has larger surface areas, enhancing the interaction between electromagnetic waves and the material, thereby contributing to improved absorption performance. A similar law was found in FCIP and SCIP-reinforced nonwoven materials. In addition, FCIP may introduce multi-scale effects, which are crucial for achieving absorption across different frequency ranges. By judiciously designing the morphology and weight ratio of CIP in CIP-reinforced nonwoven fabrics, an enhancement in absorption performance can be achieved over a broader frequency range. Compared with SCIP-reinforced nonwoven fabrics, FCIP-reinforced nonwoven fabrics can improve EMW absorption performance over a broader frequency range. FCIP exhibits significant orientation effects in composite materials, providing potential superior EMW absorption performance. SCIP has better flowability and can be more evenly dispersed in composite materials, contributing to the overall uniformity of the material, which is crucial for the stability and reliability of mechanical properties.

## 2. Experiments

### 2.1. Materials and Reagents

Aqueous polyurethane (PU, YC-601C, 43% concentration) and thickener (YC-100B) were supplied by Anhui Yuanchen New Materials Technology Co., Ltd. (Hefei, China). Spherical and flake-like CIP were purchased from Hebei Borsam Metal Materials Co., Ltd. (Xingtai, China). The dispersant was sourced from Guangzhou Aohexin New Materials Co., Ltd. (Guangzhou, China). The 1 mm thick polyester needle-punched nonwoven fabric (120 g/m^2^) was obtained from Yiwu Piccolo Electronic Commerce Co., Ltd. (Yiwu, China). Purified water was prepared in the laboratory.

### 2.2. Preparation of CIP/PU/PET Composites

Two different morphologies of CIP, spherical and flake-like, with average sizes of 4.71 μm and 4.75 μm, respectively, were used. These two types of CIP were mixed with PU in ratios of 1:2, 1:1, and 2:1. The control group consisted of PU/PET composites without the addition of CIP, as shown in Table 1.

The sample preparation process for testing the absorption performance of CIP-modified nonwoven materials using the arch method is as follows: First, PET nonwoven fabric was washed with ethanol and deionized water to remove impurities such as oil and dust from the surface of the substrate. Afterward, the fabric was placed in an oven at 60 °C for 12 h and allowed to cool naturally. Next, PU and 1 wt.% dispersant were added to a 250 mL beaker. The beaker was secured to the top of an ice bath circulating bucket using homemade foam boards. A high-speed variable-speed mixer was used to stir the contents at 500 r/min for 5 min. The stirring speed was then increased to 1000 r/min, and CIP was slowly added while stirring. Half of the water was added slowly, and the mixture was stirred for 30 min to ensure thorough dispersion of the CIP. The remaining half of the water and 0.1 wt.% thickener were slowly added, and the mixture was stirred for an additional 20 min. The stirring speed was then gradually reduced until it stopped, resulting in the CIP/PU solution.

The solution was poured into a mold, and when the solution naturally flowed evenly, the pre-cleaned and dried nonwoven fabric was laid flat in the mold. A scraper was used to gently coat the surface of the nonwoven fabric to ensure the uniform distribution and complete impregnation of the solution. Finally, the mold was left to air dry for 24 h. After the nonwoven fabric had dried, the samples were immersed in water at 52 °C for 20 min. Subsequently, the fabric samples were removed from the mold and allowed to air dry naturally, and then the CIP/PU/PET composite was obtained.

The sample preparation process for testing the electromagnetic performance of CIP using the coaxial method is as follows: First, weigh a certain quantity of flake paraffin wax and CIP, according to a mass ratio of CIP/flake paraffin wax = 2:1. Second, mix both materials evenly in a mortar. Then, take the mixture and place it into a ring-shaped mold, of which the inner and outer diameters were 3.04 mm and 7.00 mm, respectively. In this way, rings with inner and outer diameters of 3.04 mm and 7.00 mm and a thickness of about 2~3 mm were made.

### 2.3. Instruments and Measurements

The microstructure of the composites was observed using a desktop scanning electron microscope (ZEISS Gemini SEM 300, Carl Zeiss AG, Oberkochen, Germany). The content of each element in the composites was quantitatively analyzed using an EDS spectrometer (X MAX-50, Oxford, UK). The structure of CIP-modified nonwoven materials was characterized using an X-ray diffractometer (Smartlab type, Rigaku Corporation, Tokyo, Japan).

The tensile properties of the composites were measured using a material testing machine (3119-609 type, Instron Corporation, Boston, MA, USA). According to the ISO 37:2005 standard [34], dumbbell-shaped samples were prepared in both transverse and longitudinal directions, with three samples for each. The tensile rate was set at 200 mm/min. The maximum tensile force and elongation at the point of fracture were calculated using Equations (1) and (2):

(1) $$\varepsilon (\%)=\frac{∆L}{L_{0}}\times 100\%$$

(2) $$\sigma \left(MPa\right)=\frac{F}{dl}$$

In the formulas, ∆*L* represents the elongation at the point of fracture of the sample, mm. *L*_0_ represents the original length of the sample, mm. *F* represents the maximum tensile force at the point of fracture of the sample, N. *l* represents the width, mm, and *d* represents the average thickness, mm, which were measured using a fabric thickness gauge (YG(B)141D type, Wenzhou Darong Textile Instrument Co., Ltd., Wenzhou, China).

To measure the complex permittivity and complex permeability of the CIP in the frequency range of 0.1 GHz to 18 GHz, CIP particles were mixed with paraffin in a mass ratio of 2:1, and coaxial samples with an outer diameter of 7 mm and an inner diameter of 3.04 mm were prepared. Measurements were conducted using a vector network analyzer (AV3672C, China Electronics Instrument & Meter Co., Ltd., Qingdao, China). The specific testing steps are as follows: place the CIP sample in the coaxial testing fixtures, ensuring the sample has been fixed uniformly in the fixtures; calibrate the coaxial cables to ensure the testing system can accurately measure; utilize the testing system to measure and record the permittivity and permeability of the CIP sample; and finally, calculate dielectric loss tangent, magnetic loss tangent, impedance matching value, and attenuation constant, and compute reflection loss between 0.1 GHz to 18 GH in the thicknesses of 1, 2, 3, 4, and 5 mm.

The electromagnetic wave absorption performance of the modified nonwoven materials, of which the size was 30 cm × 30 cm, was tested according to the arched frame method on an arched frame testing system (The 41st Research Institute of China Electronics Technology Group Corporation, Qingdao, China). The testing frequency range was set from 12 GHz to 18 GHz. The specific testing steps are as follows: Firstly, connect the vector network analyzer to the computer and the arched frame testing system, and initiate the system for preheating. Next, follow the prompts on the computer to input parameters and calibrate the testing system. Once the calibration is complete, place the standard sample on the sample holder, ensuring it maintains the standard temperature between 20~26 °C. Then, follow the specified procedures on the computer to measure the reflection loss of the standard board. After completing the measurement, remove the standard board and replace it with a sample to be tested. Successively, test each sample according to the requirements.

## 3. Results and Discussion

### 3.1. Surface Morphology

**Figure 1:**
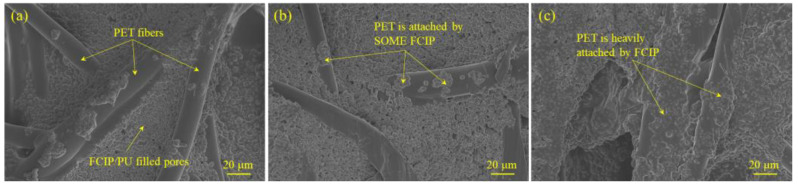
The morphological images of SCIP/PU/PET composites with different mass ratios: (**a**) S-1; (**b**) S-2; and (**c**) S-3.

Figure 1 displays the microstructure of the SCIP/PU/PET composite material. From Figure 1a,b, it is evident that SCIP is well dispersed in the aqueous PU. As the mass ratio of SCIP increases, the distance between particles becomes closer, but there is no significant aggregation. Additionally, SCIP/PU fills the gaps between the fibers, indicating good compatibility between SCIP and PU, resulting in a homogeneous composite system. With the increase in the mass ratio of SCIP, more SCIP/PU adheres to the surface of the fibers. From Figure 1c, it can be seen that when the ratio of SCIP to PU is 2:1, most of the fiber surface is covered by SCIP/PU, leading to an increased roughness. The uniform dispersion of SCIP helps to avoid electromagnetic coupling and aggregation, reducing reflection and transmission losses while increasing absorption losses.

**Figure 2:**
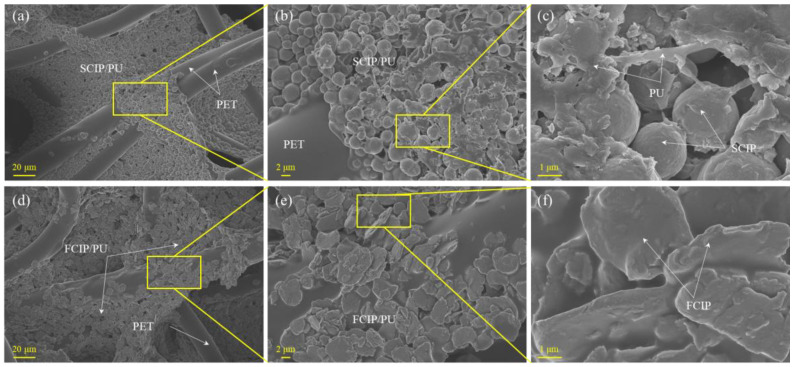
Microstructure images at different magnifications: (**a**) 500×, (**b**) 2000×, and (**c**) 10,000× of S-3; (**d**) 500×, (**e**) 2000×, and (**f**) 10,000× of F-3.

To further observe the adhesion of CIP/PU, Figure 2 presents the microstructure images of S-3 and F-3 at different magnifications. It can be observed that in Figure 2a,d, CIP adheres significantly to the surface of the fibers and fills the gaps between the fibers. In Figure 2b,e, CIP is uniformly dispersed in PU and adheres to the fibers without significant aggregation, which is favorable for electromagnetic wave absorption. Figure 2c,f show that SCIP has regular spherical particles with a smooth surface, which makes it difficult for spherical aggregates to form and ensures uniform distribution in PU. On the other hand, FCIP has an irregular, flake-like particle morphology, and it exhibits significant planar anisotropy, which means it displays different magnetic properties in different directions. This characteristic has the potential to influence the magnetic distribution within the composite, ultimately leading to optimized magnetic performance. The anisotropic nature of FCIP particles can be strategically utilized to enhance the composite material’s overall magnetic properties, making it more effective for electromagnetic wave absorption.

### 3.2. XRD Analysis

**Figure 3:**
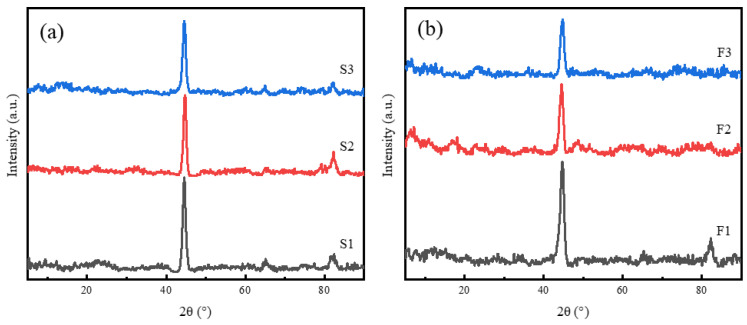
XRD spectra: (**a**) SCIP/PU/PET; (**b**) FCIP/PU/PET.

The X-ray diffraction (XRD) tests conducted on CIP-modified polyurethane (PU) matrix nonwoven materials revealed some significant features. Figure 3 is the X-ray diffraction (XRD) pattern of CIP/PU/PET composite materials with different mass ratios of CIP-loaded composite materials. The SCIP-reinforced composite material exhibits three significant strong peaks at 2θ = 44.50°, 64.98°, and 82.22°, corresponding to the (110), (200), and (211) crystal planes, respectively. Consistent with the standard card (JCPDS No.06-0696), the SCIP in the composite material is a cubic crystal. The introduction of PU and PET nonwoven materials does not change the crystal structure of SCIP. In addition, no diffraction peak of PU was found in the SCIP-reinforced composite material, as the intensity of the diffraction peak of PU observed near 2θ = 20° is weak, and the full width at half maxima (FWHM) is large [35], which is covered by the strong diffraction peak of carbonyl iron crystals. As the FCIP content increases, the diffraction peak intensity of carbonyl iron in FCIP-reinforced composite materials gradually decreases, possibly due to the exothermic effect of polyurethane polymerization reaction on FCIP, which damages the original lattice system and increases internal defects.

### 3.3. Mechanical Performance Analysis

**Figure 4:**
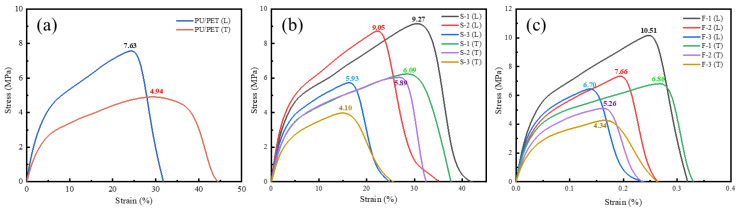
Tensile stress–strain curves: (**a**) control group PU/PET; (**b**) composites with different SCIP contents; and (**c**) composites with different FCIP contents.

The mechanical performance (tensile strength and elongation at break) of composites with different ratios was tested using a universal material testing machine, as shown in Figure 4. Figure 4a displays the stress–strain curves for the transverse tensile (perpendicular to the combing direction, T) and longitudinal tensile (parallel to the combing direction, L) of PU/PET. It can be observed that the material exhibits anisotropic tensile properties in both directions. The longitudinal tensile strength is higher, reaching 7.63 MPa, while the transverse tensile strength is 4.94 MPa. This is because the PET nonwoven material requires combing of the fibers before needling consolidation, which orients the short fibers along the combing direction, resulting in higher tensile stress in that direction.

Figure 4b shows the stress–strain curves for SCIP/PU/PET composites, with better tensile performance in the longitudinal direction. As the SCIP content increases, the tensile performance of the composites decreases. S-1 exhibits the highest tensile strength, reaching 9.27 MPa. In comparison with Figure 4a, the tensile performance of the composite materials first increases and then decreases. This is because the presence of SCIP particles provides more nuclei for the crystallization of PU hard segments, improving their crystallinity. However, when there is an excess of SCIP particles, they noticeably occupy the crystalline regions of the PU matrix, impeding crystallization and leading to a decrease in tensile performance.

Figure 4c shows the tensile performance curves of FCIP/PU/PET composites prepared with different FCIP contents. With an increase in FCIP content, the tensile performance of FCIP/PU/PET composites decreases. Since FCIP has poor flowability and is prone to fiber orientation on the surface, when the FCIP content is low, it promotes the formation of PU crystalline regions. Thus, the best tensile performance is achieved when the FCIP: PU ratio is 1:2. However, when the FCIP: PU ratio is further increased, the relatively higher FCIP content limits the further formation of PU crystallinity, resulting in reduced tensile performance.

**Figure 5:**
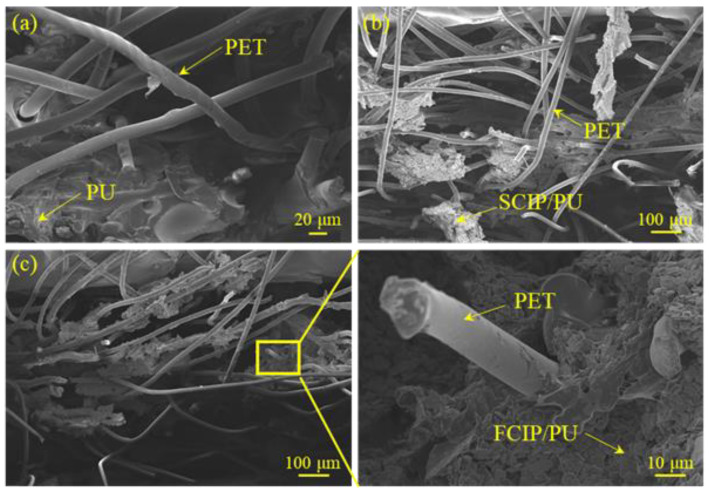
Tensile fracture surfaces: (**a**) control group PU/PET; (**b**) S-3; and (**c**) F-3.

Figure 5a presents the tensile fracture surface of PU/PET, encompassing solely PU and PET materials. Under tensile stress, the PU matrix undergoes brittle fracture and separates from the PET. In Figure 5b, the cross-section of S-3 is displayed. The tensile fracture analysis reveals a conspicuous coating of PET with SCIP/PU, effectively filling the gaps between PET fibers. As a result, stress is transmitted more efficiently and uniformly among the PET fibers. Even on the torn PET fibers, a considerable amount of SCIP/PU adheres, underscoring the influence of both PET fibers and SCIP/PU on the tensile performance of SCIP/PU/PET materials. Moving to Figure 5c, the tensile fracture section of F-3 reveals a complex intertwining of fibers with a notable presence of FCIP/PU adhered to them. Upon closer inspection of the F-3 section, the magnified view discloses a multitude of fibers that have been pulled out, resulting in the appearance of fibers or voids on the composite’s fracture surface. The matrix surface exhibits a scaly, uneven plastic fracture pattern. This observation indicates the formation of a strong interfacial bond between the fibers and the matrix, enabling the matrix to effectively transmit the load to the fiber material, which can efficiently bear the load.

Therefore, the addition of CIP can enhance the mechanical properties of nonwoven fabric. This is because CIP itself possesses high hardness and strength, and in composite materials, CIP can form a uniform dispersed structure, imparting better overall mechanical performance to the material. Additionally, CIP dispersed in PU can create an effective support structure within the nonwoven materials, aiding in the dispersion and transmission of external stress. This support structure can impede the expansion of cracks, thereby improving the tensile strength of the nonwoven materials. Furthermore, the filling effect of CIP in the composite can increase the mass density. This filling effect allows the nonwoven substrate to more uniformly transmit and distribute forces under stress loading.

### 3.4. Electromagnetic Absorption Performance and Electromagnetic Parameters of CIP

#### 3.4.1. Electromagnetic Absorption Performance of CIP

To assess the electromagnetic wave absorption performance of CIP, the powder was mixed with paraffin in a mass ratio of 2:1. The experimentally obtained complex permittivity (*ε_r_*) and complex permeability (*μ_r_*) were then used in Equations (3) and (4) to calculate the reflection loss (R_L_) of the two powder samples [3]. This study investigated the electromagnetic wave absorption performance of the two powders:

(3) $$R_{L}\left(dB\right)=20log\left|\left(Z_{in}-Z_{0}\right)/\left(Z_{in}+Z_{0}\right)\right|$$

(4) $$Z_{in}=Z_{0}\sqrt{\mu_{r}/\varepsilon_{r}}\tanh\left(j\frac{2\pi fd\sqrt{\mu_{r}\varepsilon_{r}}}{c}\right)$$

where *f* represents the electromagnetic wave frequency, *Z*_in_ is the input impedance, *Z*_0_ is the impedance of free space, *d* is the thickness of the absorbing material, and *c* is the speed of light.

**Figure 6:**
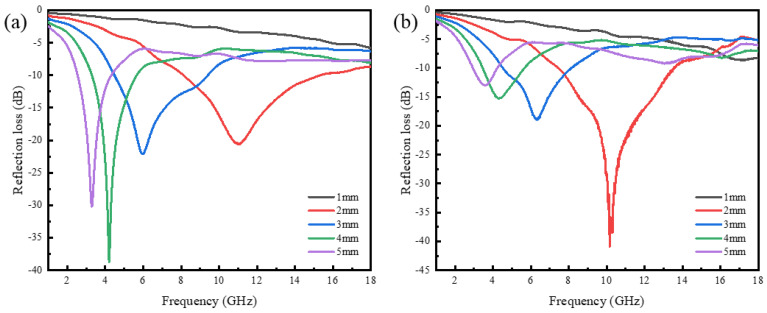
Reflection loss of different CIPs: (**a**) SCIP; (**b**) FCIP.

The calculation results are shown in Figure 6. According to Equation (3), when RL is less than −10 dB, more than 90% of the incident electromagnetic waves are absorbed, which can be referred to as effective absorption. It can be seen that when the absorber thickness was 1 mm, neither of the two powders exhibited effective absorption. When the absorber thickness was 2 mm, both types of powders exhibited a maximum range of effective absorption in the vicinity of 10 GHz. In particular, FCIP achieved the minimum reflection loss peak. The minimum reflectance loss value for FCIP was −40.93 dB at 10.16 GHz, corresponding to an effective absorption bandwidth of 6.08 GHz (7.47~13.55 GHz). It is evident that FCIP exhibits better electromagnetic wave absorption performance compared with SCIP. This is because, according to the Kittel equation, the anisotropic shape of magnetic materials, such as flake-like or elliptical, can potentially allow the material to exceed Snoek’s limit and achieve high permeability at high frequencies, thereby altering the impedance matching performance of carbonyl iron [36].

#### 3.4.2. Electromagnetic Parameters of CIP

To further understand the differences in the microwave absorption performance of different types of CIPs, the fundamental electromagnetic parameters, namely permittivity and magnetic permeability, of the two materials were analyzed. *ε*′ and *ε*″ represent the real and imaginary parts of the permittivity, respectively, indicating the storage and loss capability of electrical energy. *μ*′ and *μ*″ represent the real and imaginary parts of the magnetic permeability, indicating the storage and loss capability of magnetic energy [37].

**Figure 7:**
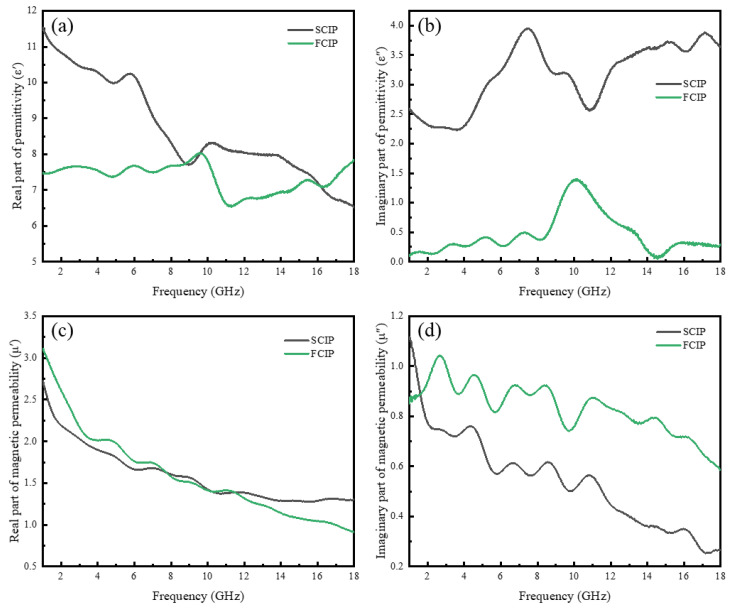
Permittivity and magnetic permeability: (**a**) real part of the permittivity; (**b**) imaginary part of the permittivity; (**c**) real part of the magnetic permeability; and (**d**) imaginary part of the magnetic permeability.

Figure 7 illustrates the electromagnetic parameters of the two different shapes and sizes of CIPs. As shown in Figure 7a, the *ε*′ value of SCIP is generally higher than that of FCIP. FCIP exhibits slight fluctuations with an *ε*′ value of around 7. As seen in Figure 7b, for *ε*″, SCIP displays strong resonance in the frequency range of 4~12 GHz, followed by a nearly constant value of approximately 3.5. FCIP maintains *ε*″ values within the range of 0~1.5. Typically, there are four main types of polarization losses, namely ion polarization, electron polarization, dipole polarization, and interface polarization, originating from relaxation and resonance mechanisms [38,39]. Ion and electron polarization can be excluded, because they often exist in 10^3^~10^6^ GHz [39]. Compared with dipole polarization, interface polarization leads to significant changes in the dielectric constant [40]. Therefore, the peaks observed in SCIP around 7 GHz and 17 GHz, and in FCIP around 7.2 GHz and 10 GHz are attributed to interface polarization, which is caused by the interface between carbonyl iron and paraffin. The fluctuations observed in SCIP around 3 GHz, and in FCIP around 3.2 GHz and 5.2 GHz are attributed to the dipole polarization, caused by polarized CIP particles.

Figure 7c shows that the *μ*′ values of all samples decrease with increasing frequency, with initial values between 2.7 and 3.3. According to Figure 7d, the *μ*″ values of all samples remain relatively stable in the high-frequency region, with FCIP having the highest *μ*″ value compared with SCIP. The magnetic loss of electromagnetic absorbing materials to electromagnetic waves mainly comes from domain wall displacement, hysteresis, eddy current loss, and natural resonance [41,42]. Domain wall displacement occurs only in multidomain magnetic materials and typically occurs in the range of 1–100 MHz [43]. Hysteresis comes from irreversible magnetization and can be ignored under weak external magnetic fields [44]. For carbonyl iron, due to eddy current losses caused by electromagnetic waves, the imaginary part of magnetic permeability exhibits a decay behavior towards high frequencies [45]. Meanwhile, several resonance peaks appeared on the imaginary part curves of the two types of CIP magnetic permeability. The existence of multiple resonance peaks mainly comes from natural resonance and exchange resonance in the gigahertz range [46]. The frequency of exchange resonance is higher than that of natural resonance; therefore, resonance peaks in the frequency range below 10 GHz are caused by natural resonance, while resonance peaks in the frequency range of 10–18 GHz should be attributed to exchange resonance [47].

**Figure 8:**
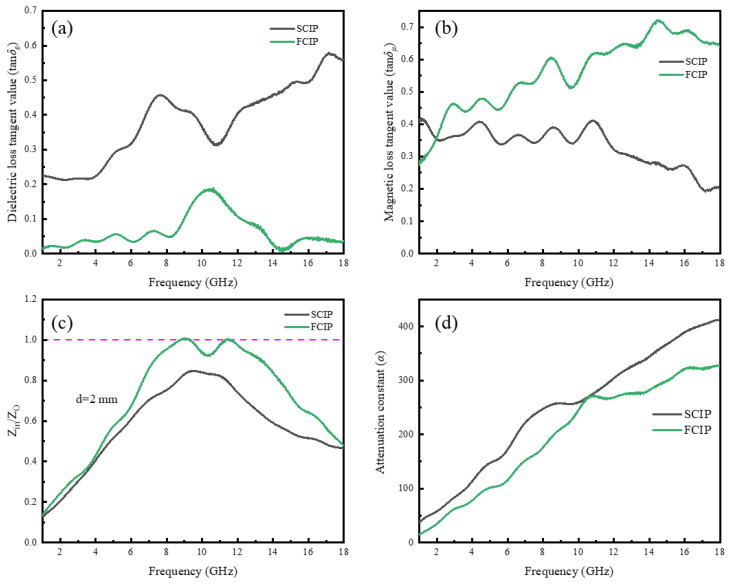
Electromagnetic parameters: (**a**) dielectric loss tangent; (**b**) magnetic loss tangent; (**c**) impedance matching value; and (**d**) attenuation constant.

The dielectric loss tangent (tan*δ_ε_*) and magnetic loss tangent (tan*δ_μ_*) are two commonly used physical quantities to describe the absorptive properties of materials. SCIP exhibits relatively high tan*δ_ε_* throughout the entire frequency range (Figure 8a), mainly due to its inherently higher *ε*″. FCIP shows strong tan*δ_μ_* values in the high-frequency region (Figure 8b), which is crucial for electromagnetic wave absorption. Results from the dielectric loss tangent tan*δ_ε_* and magnetic loss tangent tan*δ_μ_* can be used to derive impedance matching and attenuation constants, both of which are relevant to the absorptive properties of materials. FCIP has an impedance matching value close to 1 (Figure 8c), meeting the necessary conditions for optimal impedance matching. Correspondingly, FCIP achieves a minimum reflection loss of −40.93 dB at 10.16 GHz. The overall attenuation constant *α*, which reflects the intrinsic dissipation capacity of electromagnetic waves within the absorber, is not significantly different between FCIP and SCIP.

### 3.5. Electromagnetic Wave Absorption Performance of CIP/PU/PET Composites

**Figure 9:**
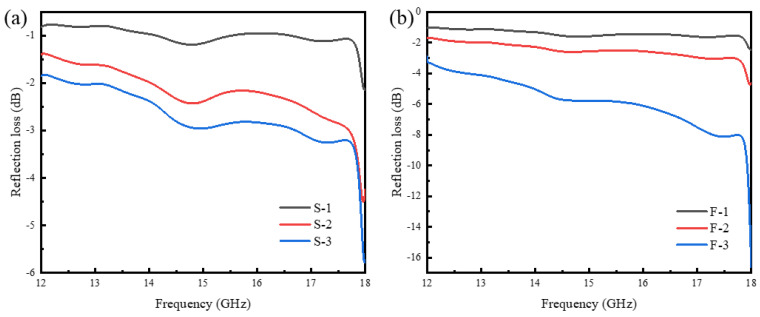
Reflection loss of CIP/PU/PET composites: (**a**) SCIP; (**b**) FCIP.

The absorption performance of CIP/PU/PET composites with a thickness of approximately 1 mm in the range of 12~18 GHz is shown in Figure 9. It can be observed that the reflection loss (RL) values of both composites increase with an increase in the mass ratio of CIPs. The RL values of the FCIP-reinforced composite are significantly higher than that of the SCIP-reinforced composite. For F-3, the minimum RL value reaches −17 dB at a frequency of 18 GHz. This value is even better than the calculated RL value of CIP at the thickness of 1 mm. The anisotropic shape of flake-like magnetic materials is further strengthened when adhering to the surface of PET fiber material, resulting in enhanced high-frequency magnetic permeability of the powder. This enhances the impedance matching performance of CIP/PU/PET composites, allowing them to break the original 2 mm thickness limit and achieve peak effective absorption at a thinner 1 mm thickness. Thus, the addition of fibrous skeleton materials holds the potential to enhance the impedance matching of flake-like magnetic particles, forming wearable and microwave absorption composites.

### 3.6. Discussion

In this study, we prepared electromagnetic wave-absorbing composites by utilizing two different morphologies of CIP (spherical and flake-like) and mixing them separately with water-based polyurethane to create different ratios of impregnation solutions. These solutions were then used to prepare composites that were impregnated into polyester needle-punched nonwoven fabric.

Regarding mechanical properties, the study evaluated the tensile strength and elongation at the break of composites with different ratios. The results indicate that FCIP/PU/PET composites exhibit better tensile performance in the longitudinal direction. As the FCIP content increases, the tensile properties of the composite decrease. The composite shows optimal tensile performance when the FCIP:PU ratio is 1:2. However, further increasing the FCIP:PU ratio restricts the further formation of PU crystallinity, leading to a reduction in tensile performance.

Discussion on electromagnetic wave absorption performance reveals that FCIP exhibits the best reflection loss value around 10 GHz, reaching a peak of −40.93 dB. This is attributed to the flake-like morphology of FCIP. The peculiar shape of the magnetic material holds promise to surpass Snoek’s limit, achieving high magnetic permeability at high frequencies, thereby altering the impedance matching performance of carbonyl iron. Electromagnetic parameter analysis shows that the real part of the permittivity and permeability of FCIP is relatively low, while the imaginary parts of the permittivity and permeability of both FCIP and SCIP exhibit high fluctuations. These fluctuations may arise from electromagnetic wave energy loss caused mainly by dipole polarization, interface polarization, natural resonance, and exchange resonance in the composites.

Finally, this study tested the absorption performance of CIP-modified nonwoven fabric in the range of 12~18 GHz. The results indicate that the reflection loss of FCIP/PU/PET composites increases with the FCIP content, and the amplitude is significantly higher than that of SCIP-modified composites. This is because the peculiar shape of FCIP in the PET matrix enhances the high-frequency magnetic permeability, improving impedance matching performance, which allows the composite material to achieve effective absorption even at a thin thickness of 1 mm.

In summary, this research provides valuable information for the development of wearable and efficient electromagnetic wave-absorbing materials by investigating the impact of different morphologies of CIP on electromagnetic wave absorption performance and mechanical properties. By selecting appropriate morphologies and contents, the performance of composites can be optimized to meet the requirements of different application fields.

## 4. Conclusions

In this study, electromagnetic wave absorption composites were prepared using CIPs with different morphologies impregnated into PET. Based on the experimental results, the following conclusions can be drawn:

(1) Concentration control during the preparation process has a significant impact on the performance of the composites. Proper CIP-to-PU mass ratio and the concentration of the impregnation solution can lead to better absorption performance.
(2) SEM observations showed that CIP is uniformly dispersed in PU, forming an effective composite structure that enhances electromagnetic wave absorption performance.
(3) The impregnated composite materials exhibit higher tensile strength compared with their non-impregnated counterparts. When the CIP mass ratio is low and the PU mass ratio is high, the composite shows higher tensile strength. Conversely, when the CIP mass ratio is high, the tensile strength of the composite decreases.
(4) The morphology of CIP significantly affects the performance of the composites. When FCIP is used, the composite exhibits the best electromagnetic wave absorption performance. At a thickness of 1 mm, this composite achieves a minimum reflection loss of −17 dB within the 12~18 GHz frequency range.

## Figures and Tables

**Table 1:** Experimental design table.

No.	CIP Type	Morphology	Mass Ratio (CIP:PU)
PU/PET	-	-	0
S-1	SCIP	Spherical	1:2
S-2	SCIP	Spherical	1:1
S-3	SCIP	Spherical	2:1
F-1	FCIP	Flake	1:2
F-2	FCIP	Flake	1:1
F-3	FCIP	Flake	2:1

## Data Availability

Data are contained within the article.
